# The hallmarks of aging as a conceptual framework for health and longevity research

**DOI:** 10.3389/fragi.2024.1334261

**Published:** 2024-01-15

**Authors:** Antonio G. Tartiere, José M. P. Freije, Carlos López-Otín

**Affiliations:** ^1^ Departamento de Bioquímica y Biología Molecular, Instituto Universitario de Oncología (IUOPA), Universidad de Oviedo, Oviedo, Spain; ^2^ Instituto de Investigación Sanitaria del Principado de Asturias (ISPA), Oviedo, Spain; ^3^ Facultad de Ciencias de la Vida y la Naturaleza, Universidad Nebrija, Madrid, Spain; ^4^ Centre de Recherche des Cordeliers, Universite de Paris Cite, Sorbonne Universite, INSERM, Paris, France

**Keywords:** healthspan, lifespan, senescence, progeria, biomarkers, rejuvenation

## Abstract

The inexorability of the aging process has sparked the curiosity of human beings since ancient times. However, despite this interest and the extraordinary scientific advances in the field, the complexity of the process has hampered its comprehension. In this context, The Hallmarks of Aging were defined in 2013 with the aim of establishing an organized, systematic and integrative view of this topic, which would serve as a conceptual framework for aging research. Ten years later and promoted by the progress in the area, an updated version included three new hallmarks while maintaining the original scope. The aim of this review is to determine to what extent The Hallmarks of Aging achieved the purpose that gave rise to them. For this aim, we have reviewed the literature citing any of the two versions of The Hallmarks of Aging and conclude that they have served as a conceptual framework not only for aging research but also for related areas of knowledge. Finally, this review discusses the new candidates to become part of the Hallmarks list, analyzing the evidence that supports whether they should or should not be incorporated.

## Introduction

The aging process has sparked human interest almost from the beginning of our existence. However, its complexity has made difficult its precise understanding and therefore its adequate definition. Nowadays, some authors define aging as “the process of accumulation of consequences of life, such as molecular and cellular damage, that leads to functional decline, chronic diseases, and ultimately mortality” (Moqri et al., 2023). In this respect, it can be argued that aging is a physiological process as it occurs as an inexorable consequence of life. In addition, the origin of this process−likely associated with the molecular damage accumulated through life−explains why it is so widespread among living creatures. Thus, aging affects animals and plants, but recent studies suggest that unicellular organisms such as bacteria, protozoa and fungi can age in certain conditions as a way to improve the possibilities of survival of half of the population (Florea, 2017).

**FIGURE 1:**
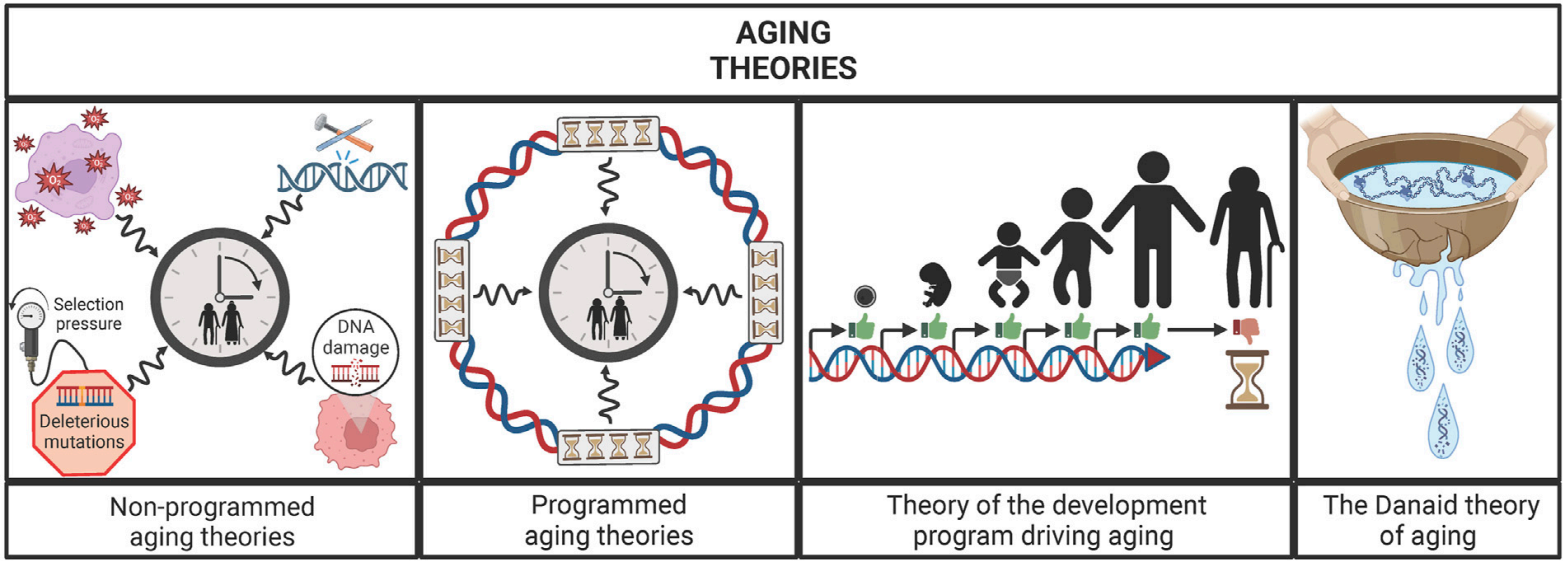
Main groups of aging theories: un-programmed aging theories, programmed aging theories, theory of the development program driving aging and the Danaid theory of aging. Un-programmed aging theories postulate that the damage accumulated through life is the engine of the aging process, while the programmed aging theories argue that a specific genetic program is what moves the “aging clock”. Between those ideas, the theory of the development program driving aging postulates that flaws of development program are the main drivers of aging. Similarly, the Danaid theory states that aging is an inherent consequence of organisms’ biology because living beings are like the cracked vessels from the Danaids, i.e., unable to hold life (water) eternally.

This widespread manifestation of aging among living beings raises a question: Has aging a biological sense? Many theories have tried to answer this question and they can be classified in two main groups: programmed or non-stochastic and non-programmed or stochastic aging theories. The main difference between them lies in the existence or not of a specific genetic program for aging. Nevertheless, this does not mean that the non-programmed aging theories deny the influence of the genome on lifespan, but that aging is not driven by a specific genetic program. In this sense, the programmed aging theories argue that aging has been evolutionarily selected due to different possible reasons. For example, some theories suggest that aging evolved as a control of population’s size and for the benefit of the species. On the other hand, the non-programmed aging theories argue that aging is an inherent consequence of living, the result of damage accumulation through life. At present, there is no definitive consensus in the scientific community on which of the two points of view is the right one (Kirkwood and Melov, 2011). Recently, a theory that lies in the middle of them has been proposed, conceiving aging as a result of “flaws” of the genetic development program. In other words, it claims that aging is not a stochastic process but it also denies the existence of a specific genetic program for aging, arguing that it is the consequence of the defects in the development program (Khokhlov, 2013; De Magalhães, 2023). A similar idea is proposed by the Danaid theory of aging, which uses the Greek myth of the Danaids as a metaphor for explaining aging. In the Greek mythology, the Danaids were women condemned to carry water for eternity in perforated vessels for murdering their husbands. In this sense, this theory argues that organisms are similar to those leaky vessels, i.e., the constraints intrinsic to their biology (holes) prevent them from holding life (water) eternally. Moreover, it also states that different taxa differ in their “permeability”, i.e., in their ability to maintain life, due to the different constraints inherent to their biology. Thus, this theory presents aging as an inexorable consequence of the biology of the taxa, being further modulated by the accumulation and selection of mutations (Wensink and Cohen, 2022) (Figure 1).

Moreover, the interest for this process has gone beyond its biological meaning, pushed by the desire for understanding its mechanisms and its causes. In this respect, this interest has grown remarkably over the last years due to the increased prevalence of aging-related diseases, which constitute some of the main causes of death worldwide. These pathologies include metabolic, neurodegenerative, cardiovascular, skin and eye diseases, articular damage and cancer, which share aging as one of their main risk factors. Therefore, further multidisciplinary studies of the aging process could enable us to understand better these diseases and, in consequence, to design improved strategies for delaying their onset and extending our healthspan. However, the complexity of the process was a major barrier to its comprehension, particularly if we consider that the knowledge about the subject was relatively unconnected and disordered. This obstacle revealed the need for a conceptual framework that could facilitate a systematic and organized approach to the problem. In this regard, in 2013 it was proposed an integrative vision of the aging process that consisted in the so-called “The hallmarks of aging”, a summary of the principal causes of aging and how they interact with each other (López-Otín et al., 2013).

### The hallmarks of aging and their history

**FIGURE 2:**
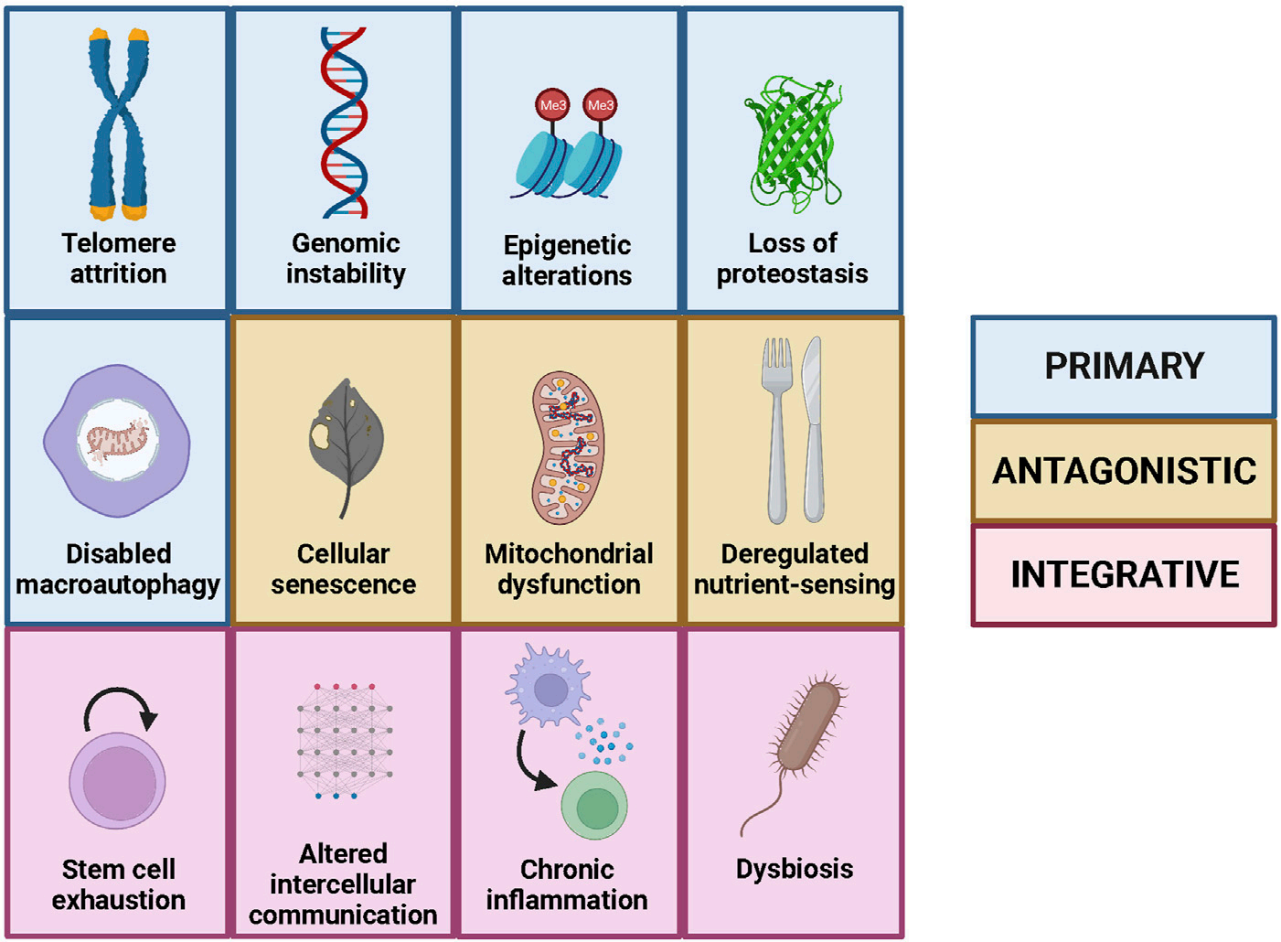
The hallmarks of aging, including the nine original ones and the three added in the second version, as well as their classification into primary, antagonistic and integrative.

The aging process is very complex and involves multiple factors that interact with each other; therefore, it was necessary to have an integrative vision that enabled a better comprehension of this quasi-universal process. In this respect, and inspired by the framework established by “The hallmarks of cancer” (Hanahan and Weinberg, 2000), López-Otín et al. proposed the existence of nine hallmarks of aging divided in three groups: primary, antagonistic and integrative (Figure 2). Furthermore, they established three criteria that a biological process must fulfil to be considered as a hallmark of aging: it has to appear during physiological aging, it has to accelerate aging when aggravated experimentally, and its experimental alleviation has to slow aging, thus increasing healthy lifespan (López-Otín et al., 2013).

The primary hallmarks of aging are intrinsically negative and comprised originally four determinants: genomic instability, telomere attrition, epigenetic alterations and loss of proteostasis (López-Otín et al., 2013). The first hallmark, genomic instability, consists in all the damage accumulated in the genome with time, including single and double strand breaks, point mutations, deletions, insertions and chromosomal alterations. Notably, it has been shown that, in mammals, species-specific rate of somatic mutation decreases with the increase in lifespan (Cagan et al., 2022). The second hallmark, telomere attrition, is a phenomenon intrinsic to the replication process, that occurs in all cells with linear chromosomes, and that limits the maximum number of divisions that each cell can undergo. This has beneficial effects, as it contributes to prevent cancer development, but also exhibits detrimental consequences because it contributes to the aging process at a cellular level. Remarkably, mice that lack telomerase, an enzyme that maintains telomere length, show premature aging (Jaskelioff et al., 2011). The third hallmark, epigenetic alterations, consists in the changes in the DNA organization and expression that do not affect to its sequence and that are caused largely by environment and lifestyle. Some of these changes, such as histone acetylation, causally contribute to the aging process as strategies used to avoid them alleviate both physiological and pathological aging in mice (Wang et al., 2021). Finally, the fourth hallmark, loss of proteostasis, refers to the decline in the homeostasis of protein synthesis, degradation and function that occurs with age. In this regard, it has been shown that the induction of protein synthesis errors leads to premature aging in mice (Shcherbakov et al., 2022).

The antagonistic hallmarks show opposing effects depending on their intensity, as they are protective at low intensity but turn into detrimental factors when they become chronic or when their intensity increases, something that occurs with age. Three of the originally proposed hallmarks of aging belonged to this group: deregulated nutrient-sensing, mitochondrial dysfunction and cellular senescence (López-Otín et al., 2013). The first one, deregulated nutrient-sensing, occurs when the cell mechanisms for determining the presence or absence of nutrients fail, so instead of protecting against nutrient scarcity they produce deleterious effects. Thus, inhibition of MTORC1 complex, a master regulator of nutrient-sensing mechanisms, increases lifespan in model organisms (Partridge et al., 2020). The second antagonistic hallmark, mitochondrial dysfunction, refers to the fact that the renewal of the mitochondria and its function deteriorates with age, leading to the accumulation of an excess of damaged mitochondria that produce detrimental products such as reactive oxygen species (ROS). These harmful species, together with the decay in the mitochondrial function, can promote the permeabilization of the mitochondria, causing inflammation and cell death (Amorim et al., 2022). However, when this occurs only at a low-grade during youth, it triggers what is called “mitohormesis”, which promotes cell survival and adaptation to stress (López-Otín et al., 2023a). Similarly, the third antagonistic hallmark, cellular senescence, has beneficial roles during youth, as it protects us from cancer and contributes to wound healing. Nevertheless, with age, senescence increases beyond physiological levels, hampering the proper function of the organism (López-Otín et al., 2023a). Actually, therapies based on senolytics, which only kill senescent cells, improve physical condition and increase lifespan in mice (Xu et al., 2018).

Finally, the integrative hallmarks are related to tissue homeostasis and originally included the two remaining hallmarks of aging: stem cell exhaustion and altered intercellular communication (López-Otín et al., 2013). The former refers to the loss of the capacity to sustain tissue renewal by stem cells due to the accumulation of damage and other factors. In this respect, cellular reprogramming ─transforming somatic cells into pluripotent stem cells by the action of four transcription factors─ has emerged as a potential strategy to recover this renewal capacity. Particularly, partial reprogramming was shown to extend lifespan in progeroid mice (Ocampo et al., 2016). The latter is related to the alterations in the communication among cells that produce erroneous, excessive or insufficient responses, which in last instance promote the aging process. A prominent example of this phenomenon is provided by blood factors with pro-aging effects, evidenced by the finding that a single transfusion of blood from old mice induces signatures of aging in young mice (Rebo et al., 2016).

These categories are not independent but hierarchically interconnected. Thus, the primary hallmarks of aging are the ones that initiate the process, as the damages produced by them accumulate with age; the antagonistic hallmarks have beneficial roles initially, but with time and as a partial consequence of the damage produced by the primary hallmarks become detrimental; and finally, the integrative hallmarks appear when the damage generated by the other groups overpass the capacity of the homeostasis mechanisms (López-Otín et al., 2013).

However, the list of hallmarks of aging was not conceived at all as a closed list and, 10 years later, the authors of the first version decided to incorporate putative new hallmarks that had arisen from the knowledge generated by the aging research community during the last decade. In this regard, three new hallmarks were finally added to the previous nine determinants of aging: disabled macroautophagy, chronic inflammation and dysbiosis (López-Otín et al., 2023a) (Figure 2). The first one belongs to the primary hallmarks category and consists of the age-associated decline of macroautophagy, a process that enables organelle renewal and therefore the correct functioning of cells. Macroautophagy had been originally considered as part of the “Loss of proteostasis” hallmark (López-Otín et al., 2013), but the recent advances in the autophagy field, such as the induction of aging features after autophagy inhibition (Cassidy et al., 2020), have reinforced the specific relevance of this process in the context of aging. For example, overexpression of autophagy-inducing proteins, such as Atg5, increases both lifespan and healthspan in mice (Pyo et al., 2013). The other two novel hallmarks belong to the integrative category and are deeply interconnected with each other. Thus, chronic inflammation or “inflammaging” is a state of low-level inflammation triggered by different agents that appears during aging, while dysbiosis is the alteration of the microbiota, which is the community of microorganisms that live in the outer surface of our body and in the inner surface of the compartments that are in connection with the exterior (López-Otín et al., 2023a). In this sense, it has been shown that increased IL-6 plasma levels are related with all-cause mortality of the aged population (Hirata et al., 2020). On the other hand, fecal microbiota transplant, an intervention designed to reverse dysbiosis, has shown to increase both lifespan and healthspan of progeroid mice (Bárcena et al., 2019).

This recent update of the hallmarks of aging was the logic consequence of the enormous knowledge generated by aging research over the last decade. In addition, and as assessed by the impressive bibliometric impact of the original work on The Hallmarks of Aging (with more than 1,000 citations per year) it is tempting to speculate that the proposal to create a conceptual framework to facilitate the advance in aging research was well received by the scientific community, as well as by other highly influential socioeconomics media (Carr, 2023; Mosbergen, 2023). Furthermore, it should be noted that the work of The Hallmarks of Aging was reinforced by posterior studies that proposed their own catalogues of hallmarks, with remarkable coincidences (Kennedy et al., 2014; Schmauck-Medina et al., 2022). Here, we have reviewed the literature citing both versions of the Hallmarks of Aging and used a small selection of highly cited articles dealing with a variety of aging-related questions to illustrate the influence of these publications on different fields of aging research.

### The hallmarks of aging as a conceptual framework

Currently, research in any field of biology generates massive amounts of information, which hampers a global vision of the matter under study. Specially, when the subject under study is complex, a systematic and integrative approach is needed, so the scientific community can take full advantage of the knowledge generated. In this regard, both the first and the second version of The Hallmarks of Aging have contributed to create a conceptual framework that has addressed the aging process in a systematic and orderly manner, facilitating aging research.

### The hallmarks as framework for investigating the foundations of aging

**FIGURE 3:**
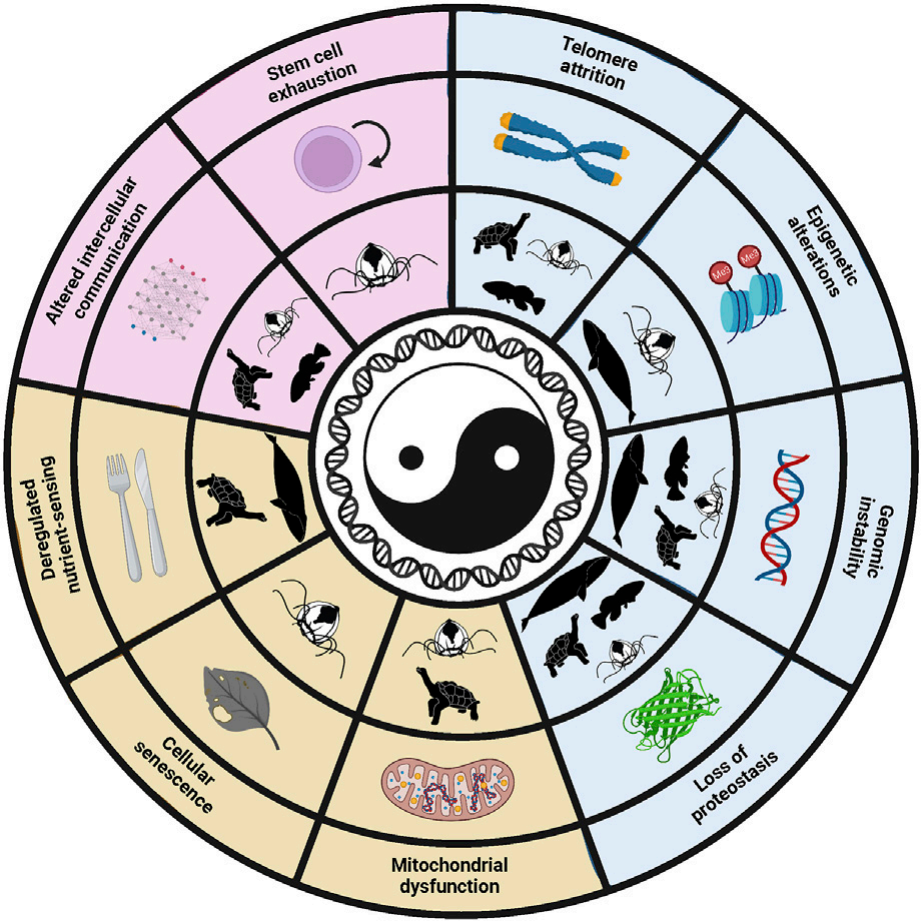
Hallmarks of aging involved in lifespan modulation of short- and long-lived species, as well as the cellular plasticity of the immortal cnidarian *T. dohrnii*. The shadows indicate which hallmarks are related with lifespan or cellular plasticity of the species. It can be appreciated that some of the hallmarks modulate the lifespan of both long- and short-lived species, pointing out that the same pathways can shorten or expand lifespan depending on the context.

Some of the best examples of how The Hallmarks of Aging set a conceptual framework for assessing the foundations of aging belong to the study of aging in long- and short-lived species. An extraordinary example of this is the outstanding work of Anne Brunet’s laboratory with African Turquoise Killifish, a fish with a lifespan of only 4–6 months in optimal laboratory conditions. To study the aging process in this species the authors used the conceptual framework provided by the Hallmarks, creating mutants for different genes associated with the first nine hallmarks and studying the functional consequences of these mutations (Harel et al., 2015). They also studied the genome of the species in a quest for insights of the genetic control of lifespan, searching for genes positively selected, comparing them with their orthologues from long-lived species and looking for their enrichment in genes related with the hallmarks (Figure 3). This group also carried out a study of variants in genes, searching for an explanation for the different lifespans among strains. Thus, the results showed that some of the variants present in the longer-lived strain, but absent in the shorter-lived, were located in genes related with the hallmarks. Moreover, some of these variants were predicted to have an impact in the protein function, suggesting a possible explanation for the differences observed in lifespan (Valenzano et al., 2015). This work represents an excellent example of how the conceptual framework provided by the Hallmarks can be used to approach the study of aging in a new model ─influencing the experimental design─ and to gain insight into the traits that control longevity, guiding the analysis of the data.

On the other hand, aging studies in long-lived species also illustrate the usefulness of the Hallmarks. For example, a study in this field analyzed the genome and transcriptome of the bowhead whale (the longest-lived mammal) in search of the determinants of its outstanding longevity. To this end, the authors searched for genes under positive selection in this species and for gene duplications and losses, noticing that some of these genes where related with the hallmarks of aging and therefore giving a possible explanation to the extraordinary longevity of this animal (Keane et al., 2015) (Figure 3). Similarly, another study examined the genome of the giant tortoises to determine possible explanations for their size, protection against cancer and longevity, with the finding that, among positively selected genes, there were some related with metabolism regulation, a hallmark of aging. Moreover, the sequence of 500 genes possibly related with aging modulation was analyzed, with the finding of significant changes −including nucleotide variants, premature stop codons and expansions− in genes related with six of the nine hallmarks. These results suggested possible explanations to the impressive longevity of these tortoises (Quesada et al., 2019) (Figure 3). A study similar to these previous ones explored the genomic keys underlying the “immortality” of *Turritopsis dohrnii*− a cnidarian able to rejuvenate after sexual reproduction. In this work, 1,000 genes related to aging, DNA repair and other relevant pathways were selected to search for variants and amplifications, and genomic differences related with all nine hallmarks of aging were found (Figure 3). Thus, the Hallmarks provided a framework for the analysis of the data and propose explanations for the extreme cellular plasticity of this species, as well as for the extreme longevity of the bowhead whale and the giant tortoises (Pascual-Torner et al., 2022).

Likewise, the Hallmarks served as a scaffold for the analysis of the results from research on aging across multiple species. An example of this is a very recent study aimed to uncover the longevity mechanisms across species and their relation to aging. For that purpose, the authors carried out an RNA-seq analysis from samples of different tissues across 43 mammalians which showed that some of the longevity mechanisms, biomarkers and lifespan-extending interventions were connected to the hallmarks of aging. For example, significant up-regulation of DNA repair and down-regulation of protein degradation were observed, which are related with genomic instability and loss of proteostasis, respectively. Moreover, the age-related transcriptomic changes shared among mammals were related with hallmarks such as mitochondrial dysfunction, cellular senescence, and chronic inflammation (Tyshkovskiy et al., 2023).

The research on cellular senescence has also reinforced the Hallmarks as a conceptual framework. For example, some studies that described transcriptomic marks conserved among senescent cells and specific marks linked to senescence-inducing stress, found that the principal gene ontology pathways altered in senescent cells were “chromatin organization” and “DNA repair”, which are associated with epigenetic alterations and genomic instability, respectively (Hernandez-Segura et al., 2017). These results supported the idea of the interconnection of the hallmarks, which was already a key part of the Hallmarks conceptual framework.

Also, the use of The Hallmarks of Aging as a conceptual scaffold has informed the experimental design of different research works, such as those showing that hyper-long telomeres do not have deleterious effects in mouse health but increase lifespan and healthspan. To support these conclusions, the study focused on some of the hallmarks of aging and checked the maintenance of telomere length with age (telomere attrition), the levels of senescent cells, the levels of DNA damage (genomic instability) and the occurrence of mitochondrial dysfunction. Notably, all these hallmarks of aging were alleviated in these mice with hyper-long telomeres (Muñoz-Lorente et al., 2019). It should be noted that there is an increasing body of scientific evidence indicating that telomere attrition is not the only cause of telomere dysfunction, which can occur regardless of the telomere length (Hewitt et al., 2012; Anderson et al., 2019; Victorelli et al., 2019).

Likewise, the studies on aging and epigenetics were not only influenced by the Hallmarks but have also contributed to demonstrate the interconnection between them and to suggest new hallmarks of aging that were incorporated in the second version of this work. For example, it has been reported that stem cell exhaustion is connected to epigenetic alterations and, in last instance, to mitochondrial dysfunction, based on the finding that during aging the blockage of the export of acetyl-CoA from the mitochondria produces chromatin changes that lead to a loss of mesenchymal stem cell pluripotency (Pouikli et al., 2021). Moreover, recent works have described the interconnection between the epigenetic alterations associated with age and cellular senescence. In this sense, a study has shown that epigenetic changes during aging in human mesenchymal progenitor cells reactivate the expression of *PSG* −a placenta specific gene− which drives cellular senescence and functions as a biomarker of aging (Liu et al., 2022). Finally, other studies have proposed the connection between epigenetic alterations and chronic or sterile inflammation, an emergent hallmark of aging that has been now included in the second version of the Hallmarks (Benayoun et al., 2019; López-Otín et al., 2023a). Therefore, the Hallmarks have not only helped to inform the analysis of the results from these studies, but have also benefited from them.

The Hallmarks of Aging were also used for the analysis of the results from some studies on the role of systemic factors in aging. In this line, recent works have investigated the effects of heterochronic parabiosis in mouse brain aging, conducing single-cell transcriptomic analyses of the brains from young and old mice after parabiosis. In these analyses, transcriptomic changes connected to the hallmarks were evaluated with the finding that heterochronic parabiosis generated changes in some hallmarks such as mitochondrial dysfunction, altered intercellular communication, cellular senescence and loss of proteostasis (Ximerakis et al., 2023). Another study in this field, determined the effects of heterochronic parabiosis in mice using a multi-omic approach. In this work, the authors performed RNA-seq analyses of liver samples from heterochronic and isochronic mice searching for changes in gene expression and for enrichment in gene sets correlated with the hallmarks. The obtained results showed that heterochronic parabiosis alleviates several hallmarks of aging such as mitochondrial dysfunction, chronic inflammation, telomere attrition, epigenetic alterations and cellular senescence (Zhang et al., 2023).

The Hallmarks have also served as a reference for the establishment of aging biomarkers. In this respect, some of the criteria for an ideal aging biomarker proposed by Moqri et al. are closely related to the hallmarks, specifically the mechanistic and generalizability criteria proposed by the authors. The former argues that a good aging biomarker has to reflect molecular and cellular mechanisms that underlie aging, i.e., it has to reflect or be causally connected with a hallmark of aging. For example, plasma proteomics or epigenetic clocks would fulfil this criterion as they reflect proteostasis and epigenetic alterations hallmarks. In this line, the second criterion argues that a valid aging biomarker should be applicable in different contexts (e.g., cell types, organs, species) and since the hallmarks are transversal, the biomarkers that reflect them fulfil this criterion (Moqri et al., 2023).

Beyond the previously mentioned fields of aging research, the study of The Tabula Muris Consortium should be also particularly discussed because of its relevance and for the use of the hallmarks in the analysis of its results. This exhaustive work consisted in the generation of a single-cell transcriptomic atlas across the lifespan of *Mus musculus*, using data from 23 different tissues and organs collected from six different time groups ranging from 1 to 30 months. This atlas was used to assess the transcriptional changes associated with age at the cellular type and tissue level, looking for changes associated with the hallmarks, such as mutational burden −related with genomic instability−, and resulted in the finding of other differences associated with them, such as the increase of senescence biomarkers or in the leukocyte population. Thus, this work also contributed to a better understanding of how these hallmarks are reflected at the cell type and tissue level (Almanzar et al., 2020).

### The hallmarks as a tool for rejuvenation research

In addition to the above discussed fields of aging research, the Hallmarks framework was also used in research on interventions aimed at reversing or slowing the aging process. These interventions are very diverse and go from cellular reprogramming or transposon regulation to metabolic interventions.

Among the cellular reprogramming studies, there are some prominent examples of how the conceptual framework of the Hallmarks was applied in this field. In this sense, a study in this area investigated whether partial reprogramming could alleviate or reverse the aging process, and to address this question followed an experimental design based on the hallmarks. First, it revealed that partial reprogramming *in vitro* −in progeroid mouse fibroblasts− ameliorated multiple hallmarks of aging. Then, it showed that an *in vivo* treatment consisting in cyclic induction of *Oct4*, *Sox2*, *Klf4* and *c-Myc* increased lifespan and healthspan in a progeroid mouse model. In addition, this treatment also improved some parameters tightly connected with the hallmarks, including the restoration of the histone marks involved in heterochromatin maintenance, the reduction in senescent biomarkers or the increase in the number of stem cells. Altogether, these results suggest that the amelioration of some of the hallmarks could be responsible for the lifespan and healthspan extending effects. Remarkably, to assess whether these findings were translatable to physiological aging and may be valid for other species, the *in vitro* treatment was repeated in late-passage mouse and human fibroblasts, revealing that the amelioration of the hallmarks also occurred in these cases (Ocampo et al., 2016). The analysis of the results from some studies on chemically-based cellular reprogramming was also facilitated by the framework provided by the Hallmarks. In this regard, a recent work performed gene set enrichment analysis for assessing the effectiveness of chemical cocktails in cell reprogramming, finding an enrichment in several gene sets related to the hallmarks of aging (e.g., DNA repair, inflammatory response, extension of telomeres), which allowed obtaining a global vision of how these cocktails affected the foundations of aging (Yang et al., 2023).

In relation to research on metabolic interventions, several studies have exploited the conceptual framework of the Hallmarks. For example, the effectiveness of caloric restriction (CR) targeting some hallmarks of aging promoted a study aimed to investigate the effect of methionine restriction in a mouse model of accelerated aging. The results showed amelioration in alterations in some pathways related to inflammation and DNA damage, resulting in extended lifespan and healthspan (Bárcena et al., 2018). Moreover, some studies have determined the transcriptional changes induced by CR in *Rattus norvegicus*, which contributed to explaining the mechanisms underlying the CR effects. Furthermore, CR was found to have an effect on the pathways related to canonical aging hallmarks and especially on altered intercellular communication (Ma et al., 2020).

The Hallmarks of Aging have also been useful for some seminal studies on the role of transposons in aging and the investigation of their potential as targets of anti-aging interventions. In this sense, some studies have revealed that the reactivation of LINE-1 during aging −in mouse and human− due to the loss of its repression triggers the IFN-I response, leading to sterile inflammation that promotes the mature senescence-associated secretory phenotype (SASP); hence, connecting chronic inflammation with cellular senescence and suggesting a possible trigger of the former (De Cecco et al., 2019). Moreover, the possibility of targeting these mobile elements to slow the aging process and revert some of its consequences was explored. In this respect, it was reported that the inhibition of the reverse transcriptase of LINE-1 in old mice alleviated IFN-I response and inflammation, suggesting this enzyme as a promising target (De Cecco et al., 2019). Other studies have shown that the mRNA of LINE-1 can be also used as a target due to the implication of this element in progeroid syndromes. Thus, LINE-1 is also overexpressed in a mouse model of Hutchinson-Gilford Progeria and systemic delivery of antisense oligonucleotides against LINE-1 mRNA improves histophysiology of tissues and their transcriptional profile, as well as increases the lifespan of these progeroid mice (Della Valle et al., 2022). Therefore, transposons seem to play a key role in physiological and accelerated aging, representing an interesting common target for both.

Finally, as aging research advances, the complex networks of interactions inside this process become patent, evidencing that strategies targeting only one of its components will hardly achieve a complete efficacy. In this context, several authors have proposed the use of combinatorial interventions, targeting different components of the aging process, as a better strategy to delay or alleviate aging. In this sense, a recent review has described the use of this type of strategies, pointing out how the Hallmarks of Aging have contributed to the development of these interventions. Particularly, describing the interactions inside the hallmarks and among them have facilitated the design of these interventions, revealing how targeting different hallmarks or components of them could lead to synergistic effects (Parkhitko et al., 2023).

### The hallmarks beyond aging research

The Hallmarks of Aging have also served as an organizing tool for studies in other fields related to the aging process such as progeroid syndromes, age-related diseases, autophagy, stress tolerance, exercise or even COVID-19.

The progeroid syndromes are considered a form of accelerated or pathological aging that mimics a significant part of the characteristics of the physiological aging process. Thus, the conceptual framework provided by the Hallmarks of Aging served as an “scaffold” for the establishment of the hallmarks of progeroid syndromes. These progeroid hallmarks are also composed by nine determinants that correlate with the ones from physiological aging (Carrero et al., 2016), thus contributing to the establishment of a systematic and integrative approach to the study of this rare group of diseases. Moreover, the Hallmarks were also present in studies of the mechanisms underlying some of these syndromes. This is the case of works that have uncovered some of the molecular basis of the Werner syndrome, caused by a mutation in the *WRN* gene. It was known that the mutation of this gene was associated with genomic instability, a hallmark of aging, but whether and how this mutation affected other hallmarks remained unclear. Notably, this mutation produces telomere attrition and epigenetic alterations, the latter promoting premature cellular senescence, which causes exhaustion of the mesenchymal stem cell pool (Zhang et al., 2015). Thus, analyzing the effects of this syndrome on the aging hallmarks led to a possible molecular explanation for the phenotype of the patients, opening new avenues for therapeutic interventions.

The Hallmarks of Aging framework has also contributed to facilitate the comprehension of some of the mechanisms underlying pathologies for which age is a major risk factor. In this sense, the Hallmarks have inspired several studies focused on the relevance and the role of the hallmarks in these diseases. For example, it has been suggested that the polyamine spermidine has cardioprotective effects and extends lifespan in mouse models, pointing out that part of these effects are exerted by improvement of mitochondrial function, reduction of the low-grade inflammatory status, restoration of proteostasis and, especially, increased autophagy (Eisenberg et al., 2016), all of them related to the established hallmarks of aging. In this line, there is evidence of accelerated biological aging in atherosclerosis plaques, which manifest most of the hallmarks of aging since their initial stages. This suggests that cellular aging has a causal role in atherosclerosis etiology, pointing out the hallmarks as possible targets for new therapeutic avenues (Uryga and Bennett, 2016). In the field of neurodegenerative diseases, there is strong evidence of the connection between neurodegeneration and the hallmarks of aging, particularly the ones related to DNA damage and mitochondrial dysfunction. In this respect, it has been shown that both Alzheimer and Parkinson disease patients present alterations in the nine original hallmarks of aging. Moreover, other neurodegenerative diseases such as amyotrophic lateral sclerosis, ataxia telangiectasia and Huntington disease have alterations in some of the hallmarks of aging (Hou et al., 2019). Hence, the hallmarks have become possible targets for therapeutic strategies with a more holistic approach (Hou et al., 2019). In the case of kidney disease, the premature-aging observed in chronic kidney disease patients is caused by the alteration of mitochondrial function, inflammation and cellular senescence produced by uremic toxins and advanced glycation end products, suggesting that targeting these hallmarks could ameliorate the poor prognosis of this disease (Ebert et al., 2020). Skin aging has also been related with the hallmarks, particularly with cellular senescence. In this respect, accumulation of senescent cells in the skin with age has been found, as well as in some skin diseases (Toutfaire et al., 2017). Moreover, clearance of senescent cells with the senolytic compound ABT-737 improved keratinocyte proliferation and epidermal thickness in a 3D culture mimicking human skin (Victorelli et al., 2019). It has also been shown that aberrant activation of the mTOR pathway, which results in deregulation of nutrient-sensing, decreases autophagy and inhibits apoptosis in idiopathic pulmonary fibrosis, highlighting the putative role of some hallmarks of aging in this disease (Romero et al., 2016). Finally, regarding chronic inflammatory diseases, there is evidence of multiple hallmarks of aging manifesting in both early and late rheumatoid arthritis patients. However, the amelioration of these hallmarks by the use of anti-inflammatory drugs, designed to alleviate these inflammatory diseases, suggests that their alteration may be a consequence rather than a cause of this disease (Chalan et al., 2015).

Regarding autophagy research, the first version of the Hallmarks facilitated some studies that later contributed to its own actualization by the incorporation of disabled macroautophagy as a new hallmark in the second version. In this line, several works have revealed that a basal level of autophagy is essential to prevent senescence in muscle stem cells from mice. More precisely, failure of autophagy in muscle stem cells from aged or genetically manipulated mice leads to mitochondrial dysfunction and loss of proteostasis and, ultimately, to cellular senescence and stem cell exhaustion (García-Prat et al., 2016). Thus, the study of the hallmarks in this work contributed to determine the mechanisms that interconnect them and to highlight autophagy as a key process in aging.

Adequate responses to stress is one of the three categories of the hallmarks of health (López-Otín and Kroemer, 2021), which are tightly connected to the hallmarks of aging (López-Otín et al., 2023a). Hence, is not surprising that the hallmarks of aging were examined in studies in the stress tolerance field. In this sense, the genome of the tardigrade *Ramazzottius varieornatus*, which has an exceptional tolerance to environmental stress, presents amplifications and variants affecting genes related to DNA repair and telomere maintenance, functions connected with genomic instability and telomere attrition hallmarks. This suggests a possible explanation for the increased resistance to stress and lifespan of this species in comparison to other tardigrades, highlighting the connection between the hallmarks of health and aging (Carrero et al., 2019).

The connection between exercise and health has been known for a long time; and accordingly, the study of the hallmarks has reached the exercise field. In this sense, the impact of exercise on the hallmarks of aging has been described, illustrating how physical activity ameliorates the different hallmarks and hence suggesting exercise as a potent and cost-free approach to promote healthy aging without undesirable side effects (Garatachea et al., 2015; Rebelo-Marques et al., 2018). On the other hand, a very recent review has described the effects of sedentary behavior on the hallmarks of aging, using bed rest, unilateral limb suspension and spaceflight studies (Raffin et al., 2023). These studies uncovered the effects of sedentary behavior on the hallmarks, especially on loss of proteostasis and deregulated nutrient-sensing. However, there is still very little evidence for some of them such as genomic instability, telomere attrition, epigenetic alterations and cellular senescence. Moreover, the focus on muscle cells and young adults, as well as the effects of other factors −such as radiation in space flight− constitute limitations that should be overcome by future research (Raffin et al., 2023). Altogether, these studies constitute a prominent example of how the framework created by the Hallmarks set the conceptual basis for some works.

As mentioned before, the Hallmarks of Aging also contributed to COVID-19 research, highlighting the diversity and relevance of the fields impacted by this integrative vision of the aging process. This has been made evident by the connection between some hallmarks of aging and the severity of COVID-19 in the elderly, which indicates that these hallmarks play a key role in the disease and suggests the use of anti-aging strategies coupled with antivirals as a novel avenue for decreasing the severity of the infection in the elderly (Salimi and Hamlyn, 2020). In this line, some studies found that age-associated changes in gene expression in the immune cells might increase the susceptibility to COVID-19 in the elderly. Moreover, COVID-19 infection increases aging-linked upregulation of genes related to inflammation and senescence, promoting a reinforcement of these hallmarks, that are tightly related with immune response and tissue regeneration. As these two functions are essential in viral response, these findings may suggest a possible explanation for the slower recovery in the elderly (Zheng et al., 2020). Likewise, another study has shown that severity of COVID-19 infection is related to telomere length in groups of different ages. Specifically, shorter telomeres are related to more severe COVID-19 in different age groups, suggesting that telomere attrition, which leads to genome instability and cellular senescence, impairs proliferation, a process that is necessary for the recovery of the infection. Moreover, this study offers a possible explanation for the greater severity of this disease in men compared to women −who have longer telomeres− and exposes again how the hallmarks of aging can affect COVID-19 infection (Sanchez-Vazquez et al., 2021). These studies illustrate how the Hallmarks have influenced COVID-19 research, setting the basis of some studies and guiding the experimental design and the analysis of the results of others.

Finally, beyond aging-related studies, the Hallmarks have become a model for the organization of knowledge that enables an integrative, systematic and organized vision of the topic. In this regard, The Hallmarks of Aging promoted other “hallmarks” such as Hallmarks of Cellular Senescence (Hernandez-Segura et al., 2018), Hallmarks of Brain Aging (Mattson and Arumugam, 2018), Hallmarks of Health (López-Otín and Kroemer, 2021), Hallmarks of T cell aging (Mittelbrunn and Kroemer, 2021) or Hallmarks of cardiovascular aging (Abdellatif et al., 2023). Moreover, the Hallmarks have given rise to the concept of meta-hallmarks of aging and cancer, which could be defined as the common hallmarks between both processes. In this sense, these meta-hallmarks aimed to illustrate the common and different foundations of aging and cancer, providing an integrative vision of both processes and their interconnections (López-Otín et al., 2023b).

### Emerging hallmarks of aging

The Hallmarks of Aging constitute an open list that is fed by the aging research promoted in part by itself. Thus, as aging research advances, the interconnections among the hallmarks are better understood and new candidates to become part of the list arise. However, in order to be considered as a *bona fide* hallmark of aging, all proposed candidates have to meet the three criteria that define the hallmarks: to appear during physiological aging, to accelerate aging when aggravated and to slow aging if alleviated.

**FIGURE 4:**
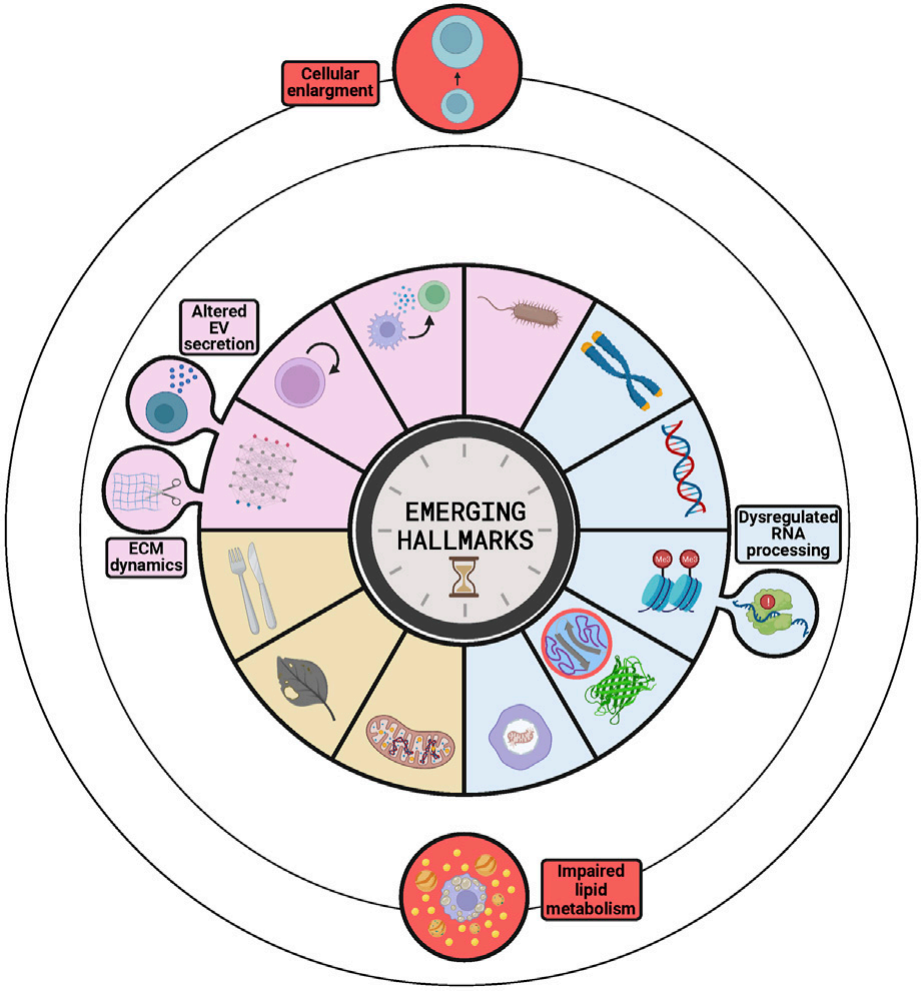
Emerging hallmarks to be considered as candidates for next versions of The Hallmarks of Aging. The buds represent those emerging hallmarks already discussed in the latest version−dysregulated RNA processing, ECM dynamics and altered EV secretion−as part of other hallmarks−epigenetic alterations and altered intercellular communication−and that are candidates to be independent hallmarks. The circles orbiting the stablished hallmarks contain additional candidates ordered by the level of evidence supporting them, stronger for impaired lipid metabolism than for cellular enlargement. The circle inside the loss of proteostasis represents intrinsically disordered proteins or regions of proteins that are becoming key elements of this hallmark.

For example, some studies have suggested the increase in cell size as an emerging hallmark of aging (Davies et al., 2022) (Figure 4). This proposal is supported by several studies indicating that an increase in cellular size occurs during aging and by the observation that increasing cell size artificially is sufficient to accelerate aging *in vitro*, while lowering cell size artificially has a rejuvenation effect *in vitro* (Davies et al., 2022). Moreover, other studies have revealed that, in mammals, there is a strong correlation between cell size of acinar cells in pancreas and salivary glands, and lifespan, so that smaller cells are associated with a longer lifespan (Anzi et al., 2018). In addition, these authors found that the naked mole rat, which lives much longer than expected according to its body size, had smaller acinar cells in the salivary gland than other animals of similar size with a much shorter lifespan (Anzi et al., 2018). However, we think that there are some drawbacks that hamper the consideration of cell size as a novel hallmark of aging. For example, some of the interventions for lowering cell size used rapamycin, whose lifespan-extending effects via mTOR inhibition were already described (López-Otín et al., 2023a). Therefore, it is arguable whether the functional improvement observed after cell size reduction with rapamycin is a consequence of the reduction on the cell size or due to any other of the multiple effects of mTOR inhibition. Furthermore, not all cell types follow this inverse correlation between cell size and lifespan, as happens with neurons that are larger in humans than in mice (Anzi et al., 2018). As pointed by some authors, the correlation observed between cell size and lifespan in some tissues could be the result of the different strategies used by the organisms to grow, hypertrophy and hyperplasia. Thus, cellular size is determined by the growth mechanism used by the organism, which would be the actual factor affecting lifespan. This possibility would be in agreement with the fact that rapid growth rates −related with hypertrophy− are associated with a short lifespan, while slow growth rates −related with hyperplasia− correlate with a long lifespan (Patra and Bardeesy, 2018).

Another candidate hallmark supported by several studies is the dysregulation of RNA processing, a process that regulates gene expression (Figure 4). This hallmark was first proposed in the Copenhagen aging meeting in 2022 (Schmauck-Medina et al., 2022) and then supported by other studies that highlighted the relevance of long non-coding RNAs (Sherazi et al., 2023) and RNA binding proteins (Varesi et al., 2023) in the aging process. These works pointed out that these elements lose their regulation during aging and how the subsequent molecular and cellular changes promote the aging process and its associated diseases (Sherazi et al., 2023; Varesi et al., 2023). However, a recent review of the field has shown that even if the dysregulation of RNA processing is a promising candidate, there is still not enough evidence of *in vivo* acceleration or amelioration of aging through interventions targeting RNA processing (Harries, 2023). Moreover, dysregulation of RNA processing was already included in the first and second version of the Hallmarks of Aging as a component of the epigenetic alterations hallmark (López-Otín et al., 2013; 2023a). Thus, future studies will determine whether dysregulation of RNA processing remains as a key component of epigenetic alterations or gains independence and constitutes a new hallmark.

Other studies have proposed impaired lipid metabolism as a possible hallmark (Figure 4), due to the evidence of its tight relation with all the established hallmarks, especially with cellular senescence and chronic inflammation (Sharma and Diwan, 2023). On the other hand, some studies have described the interaction between lipid rafts −small domains in the plasma membrane enriched in cholesterol and sphingolipids− and the different hallmarks of aging in some cell types (Zhang et al., 2022). The main function of these structures is related to signal transduction processes, as they contain signaling proteins; therefore, their alteration during aging generates erroneous signals that impact on different hallmarks (Zhang et al., 2022). However, the relationship between the changes in lipid metabolism and some of the hallmarks is still unclear, so more evidence should be necessary to consider this process as a new hallmark of aging.

Extracellular matrix (ECM) dynamics and altered extracellular vesicles (EVs) have been proposed by some studies as new hallmarks of aging. However, these processes were already included in the last version of the Hallmarks as a part of the altered intercellular communication hallmark (López-Otín et al., 2023a) (Figure 4). It can be discussed whether they should be considered as independent hallmarks or not, but it should be taken into account that the aim of The Hallmarks of Aging was to facilitate an integrative and organized vision of the aging process. Hence, to fulfil that role, the list must be both as comprehensive and concise as possible. Therefore, since ECM dynamics and EVs are essential players in intercellular communication, adding them as independent hallmarks would add unnecessary complexity to the list. Furthermore, even if some studies recognized ECM dynamics as a hallmark of aging (Schmauck-Medina et al., 2022), a recent review has shown that this process does not fulfil yet the criteria to be considered a hallmark (Statzer et al., 2023). In fact, despite data supporting the disruption of this process during physiological aging and the acceleration of aging upon its experimental disruption in *Caenorhabditis elegans* (Statzer et al., 2023), there is still no evidence of experimental amelioration of aging in mammals. On the other side, the alteration of EVs has become a promising field in aging research, after being reported that senescence is associated with an increase in EV release. In addition, administration of EVs from old or young donors has been shown to promote exacerbation or alleviation of aging, respectively. However, there is still no consensus about the changes (e.g., amount, content, effects) that occur in the EVs during aging (Manni et al., 2023). Therefore, at present the limited knowledge on the subject complicates the inclusion of altered EVs as a new hallmark of aging.

Likewise, and although it has not been proposed as an emerging hallmark, some studies have highlighted the role of intrinsically disordered proteins (IDPs) and regions (IDRs) of proteins in aging and age-related diseases. These IDPs and IDRs do not have a stable tertiary structure, which enables the protein to develop multiple functions and to interact with multiple different proteins. These properties make these proteins key players in the regulation of many processes, including those related with aging (e.g., the Yamanaka factors have multiple IDRs). In this sense, it has been found that groups of proteins related to genomic instability, epigenetic alterations, telomere attrition and cellular senescence are enriched in this type of proteins. Moreover, the aggregation of these proteins has been related with several neurodegenerative diseases, which are important age-related pathologies (Manyilov et al., 2023). Thus, the dysregulation of these proteins would be a critical component of the loss of proteostasis and a link with other hallmarks (Figure 4).

On the other hand, and as discussed before, increasing evidence suggests that telomeres can suffer other damages beyond their attrition, that also lead to their dysfunction (Hewitt et al., 2012; Anderson et al., 2019; Victorelli et al., 2019). Therefore, the telomere attrition hallmark could be expanded to encompass these new types of damages, and perhaps be renamed as telomere dysfunction. However, even though there is clear evidence that these damages increase during aging and in senescent cells, to consider them a hallmark of aging, they should be better characterized and it must be proved that their experimental aggravation and alleviation accelerates and slows aging, respectively.

Finally, it is important to remember that, despite being an open list, the incorporation of new hallmarks should be done in a very rigorous, precise and careful manner. Otherwise, they will lose their integrative, systematic and organized structure becoming impractical as a conceptual framework.

## Conclusion

The Hallmarks of Aging arose as an answer to the great amount of information generated by aging research, with the aim of creating a conceptual framework to integrate and organize the existing knowledge. The objective of the present review has been to determine the impact of the Hallmarks and address if the purpose that gave them rise was achieved. For that aim, we reviewed the literature that cited any of the two versions of the Hallmarks. The conclusion was that the first version (with the second one also following the same trend) accomplished the goal, as it influenced a vast variety of fields ranging from the different areas of aging research to other related fields. Furthermore, it also inspired other authors and served as a model for the organization of knowledge, giving rise to a wide variety of “Hallmarks” in other subjects. Nevertheless, this impact was not unidirectional, since the research promoted by the first version of the Hallmarks generated a great deal of knowledge that gave rise to the updates included in the second version. This updated version included three new hallmarks and, in spite of its recent publication, it is being highly cited and has already influenced some studies and served as a knowledge-structuring model. Therefore, it can be concluded that the usefulness of The Hallmarks of Aging in aging-related research seems undeniable. However, as any approximation it has its limitations and it should be carefully revised, considering the latest advances, to determine whether all the hallmarks are still valid and if it is necessary to include new ones. In this sense, this review has analyzed the possible emerging hallmarks that were not included in the second version and the ones that were included but as components of other hallmarks. Assuming that a conceptual framework must be as schematic and organized as possible and the still limited evidence supporting some of these candidate hallmarks, we conclude that further investigations are needed to assess if any of these proposed hallmarks should be included in the next version. Even though the current Hallmarks of Aging provide a valid scaffold for aging research, there is no doubt that, as knowledge advances, updated versions of the Hallmarks will become necessary. To this aim, we will continue working on aging research while aging will also continue working on all of us.
